# Supplementary material for: Unveiling Lovastatin’s Anti-Inflammatory Potential in Mouse’s Brain during Acute Trypanosoma cruzi Infection
Source: Biology (Basel). 2024 Apr 27;13(5):301. doi: 10.3390/biology13050301 (PMC11118176; doi:10.3390/biology13050301)
Supplement: Supplementary file 1 [file biology-13-00301-s001.zip › biology-2765748-supplementary.pdf]

Supplementary Figure

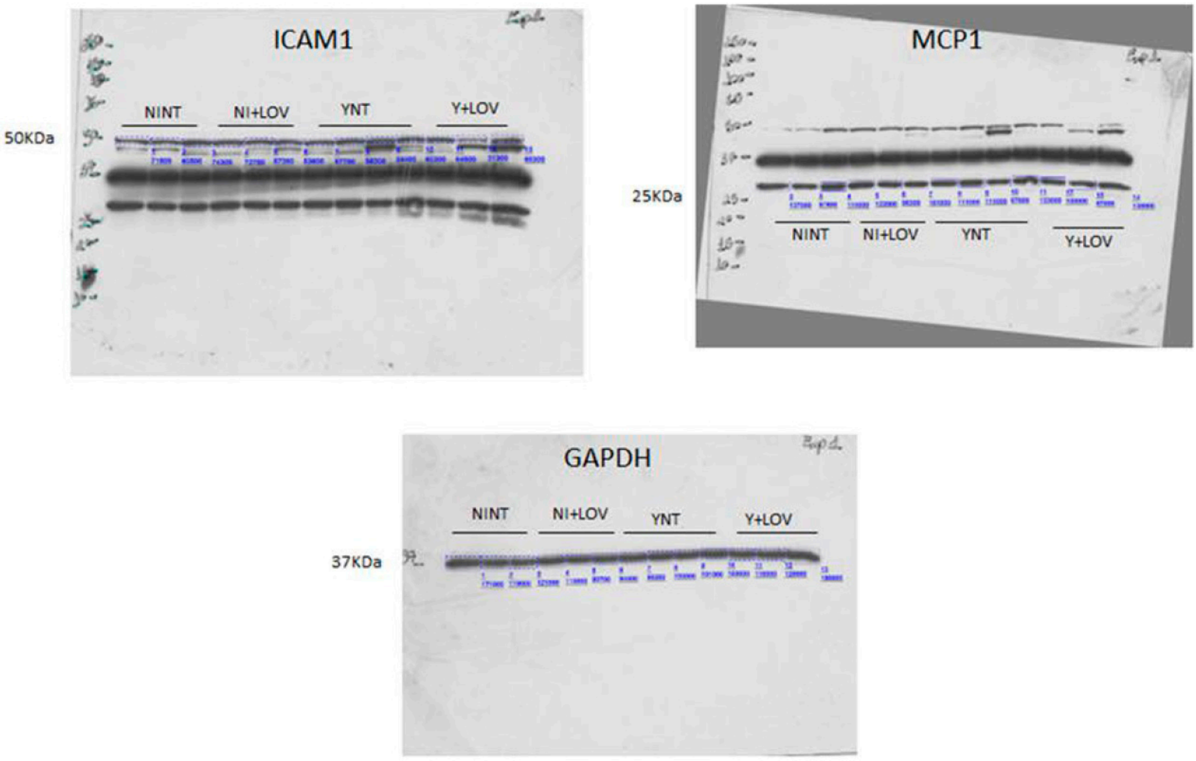

Figure S1. represents original western blot images for ICAM-1 and MCP-1, using samples from experiment 1, performed in the ssame membrane, with their loading control GAPDH.

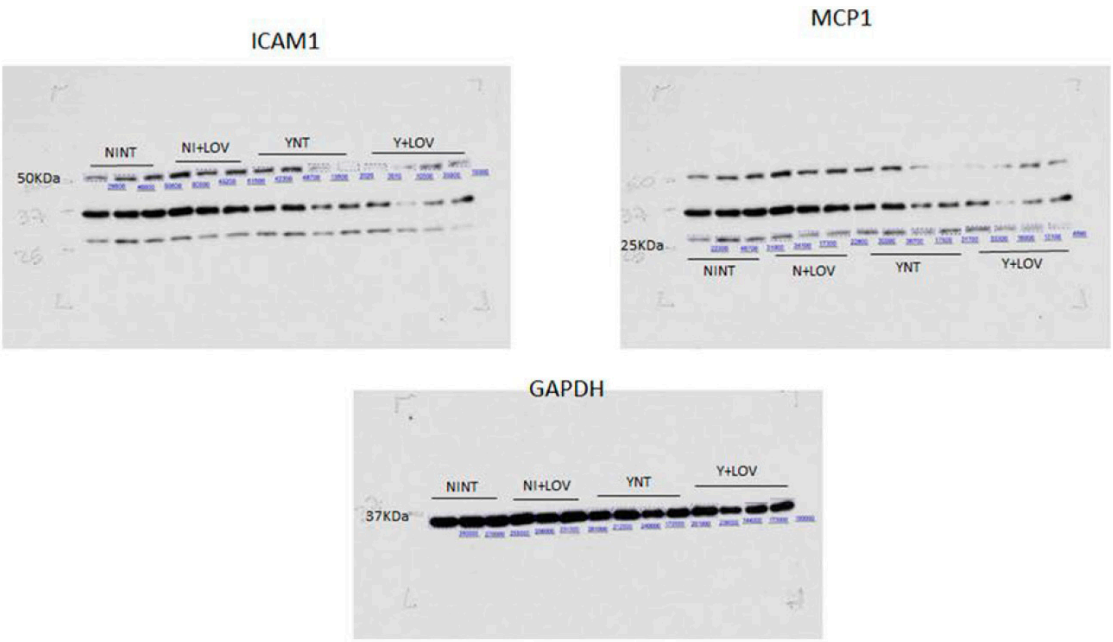

Figure S2. represents original western blot images for ICAM-1 and MCP-1, using samples from experiment 2, performed in the ssame membrane, with their loading control GAPDH.

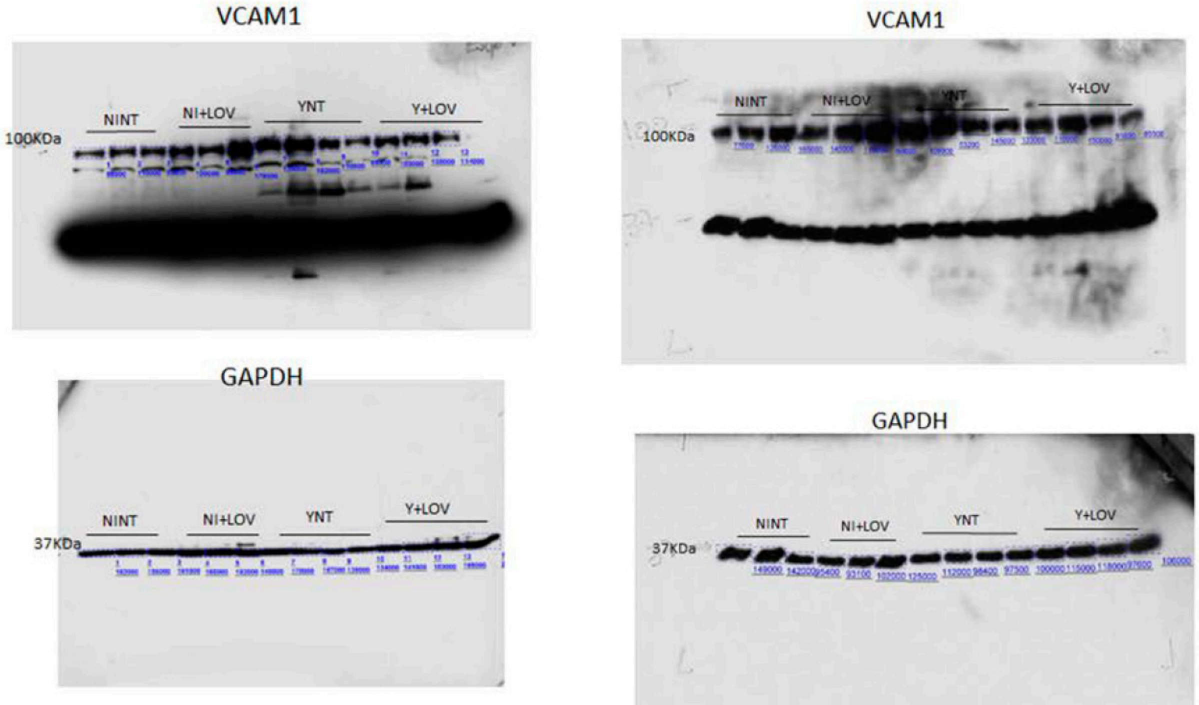

Figure S3. represents original western blot images for VCAM-1, using samples from experiment 1 and 2, with their loading control GAPDH.

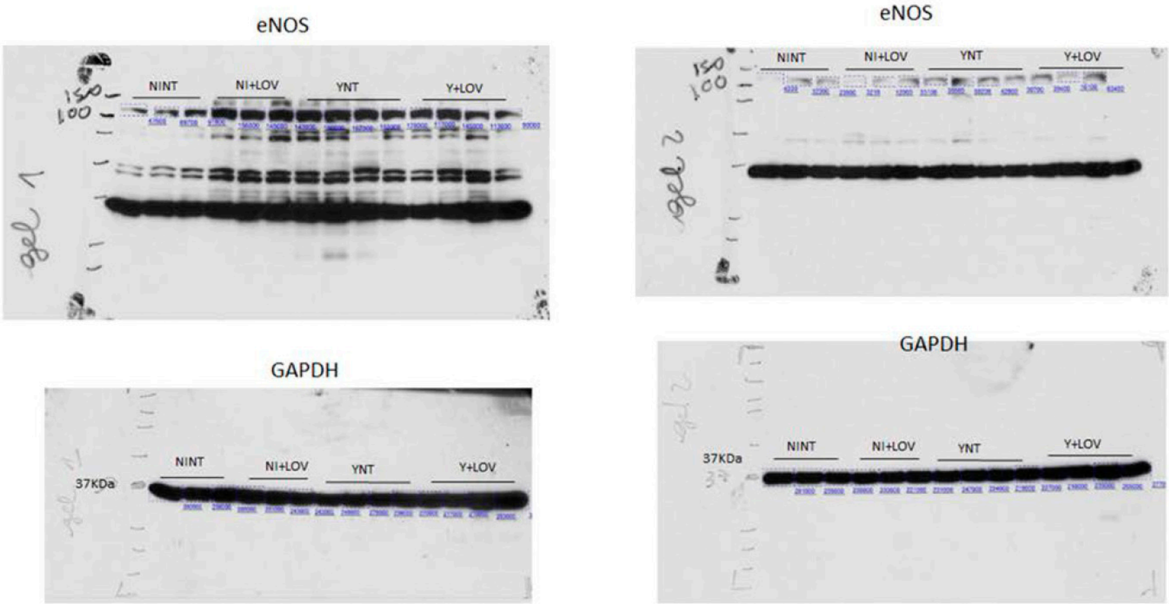

Figure S4. represents original western blot images for eNOS, using samples from experiment 1 and 2, wiith their loading control GAPDH.
